# Injury by knife crime amongst children is associated with socioeconomic deprivation: an observational study

**DOI:** 10.1007/s00383-022-05298-6

**Published:** 2022-11-28

**Authors:** John-Joe Reilly, David N. Naumann, Louise Morris, Lauren Blackburn, Adam Brooks

**Affiliations:** 1 Department of Major Trauma, East Midlands Major Trauma Centre, Queens Medical Centre, Nottingham, UK; 2 University of Birmingham, Birmingham, UK

**Keywords:** Paediatric, Knife, Stabbing, Penetrating trauma, Socioeconomic deprivation

## Abstract

**Purpose:**

Children who live in areas of socioeconomic deprivation may be at higher risk of being victims of violent crime such as knife wounds. The current study investigated whether socioeconomic disparity was associated with higher risk of knife crime.

**Methods:**

An observational study included patients aged ≤ 17 years at a UK Major Trauma Centre injured by knife trauma from 2016 to 2022. Indices of deprivation were recorded according to the zip code of residence and compared with those of all of England. These included Index of Multiple Deprivation (IMD); income; employment; education and skills; health and disability; crime; barriers to housing and services; living environment; and Income Deprivation Affecting Children Index (IDACI).

**Results:**

There were 139 patients (96% male) with median age of 16 years. When compared with the whole of England, patients had worse indices of IMD (*p* = 0.021); income (*p* < 0.001); employment (*p* < 0.001); education and skills (*p* < 0.001); health and disability; and IDACI (*p* < 0.001). There were no significant differences in indices of crime, barriers to housing and services or living environment.

**Conclusions:**

Paediatric knife injury was associated with poor socioeconomic status in multiple domains. Focussed efforts to address socioeconomic disparities should be a priority as a public health measure for vulnerable children.

## Introduction

According to a report by the World Health Organization, interpersonal violence is one of the most common causes of death and disability for young people worldwide, and this burden is more likely amongst males and those with poorer socioeconomic status [1]. Victims of violence are also more likely to become victims in the future, and therefore directed efforts to prevent this vicious cycle are required [2]. In the UK, young men appear to be the most common perpetrators and victims of knife crime [3]. Rather than simply being a matter for the police and crime investigators, knife crime also represents a public health concern due the significant effects on victims [4]. Since knife crime appears to be increasing over time in the UK [5], there is an urgent need for a focussed and effective approach to prevention that addresses the risk factors for injury.

For children, exposure to violence increases their risk of alcohol and drug abuse in adulthood [6, 7], as well as continuing victimization later in life [8]. Victims of violence in childhood have also been reported to have poorer health in adulthood, with adverse cardiovascular [9], sexual [10] and mental [11] health outcomes reported in the literature. Prevention of violent trauma amongst children is therefore paramount in reducing life-long morbidity. Tackling this issue in children is not straightforward, and there are likely to be multiple risk factors, including those within the socioeconomic domain [12]. It is timely to examine the socioeconomic risk factors for victimhood of knife crime in the UK if public health measures are to be well informed and based on evidence.

The aim of the current study was to investigate the association between socioeconomic deprivation and injury from knife crime in a sample of paediatric patients in England. We hypothesized that there would be an association between higher indices of deprivation and knife crime.

## Methods

### Study design and setting

An observational database study was undertaken to investigate paediatric patients who had been admitted to the East Midlands Major Trauma Centre in the 5 years and 3 months between December 2016 and March 2022 following injury by penetrating knife trauma. Institutional approval was granted prior to data collection (reference: 22–215C).

### Patient selection

Patients were eligible for inclusion if they were 17 years old or younger and presented to the Emergency Department (ED) following a knife injury during the study period. Patients were identified retrospectively from local hospital episode statistics according to search terms that included presentation as an assault where a knife was the weapon. Patients were excluded if they were older than 17 years, or if they had a penetrating injury that was not caused by a knife.

### Data collected

Anonymized data were stored on a password encrypted computer. The data collected included demographic characteristics (age, sex and ethnicity), timings of injury and zip codes of their place of residence. The zip codes were used to derive all of the socioeconomic parameters from the UK Government website (https://www.gov.uk/government/statistics/english-indices-of-deprivation-2019), which was compiled by the Ministry of Housing, Communities and Local Government in 2019 and accessible in the public domain. These parameters are based on the local administrative and census data, and have been updated every 3–5 years since 2000. These parameters included deciles for Index of Multiple Deprivation (IMD); income; employment; education and skills; health deprivation and disability; crime; barriers to housing and services; living environment; and Income Deprivation Affecting Children Index (IDACI). These parameters were also collected for the whole of England as a control reference point to compare the study cohort. In all cases, lower deciles indicate worse deprivation, with the 1^st^ decile being the most deprived, and 10th decile being the least deprived. Details regarding management of injuries and hospital admission were recorded. The outcomes of interest included mortality and re-admission within 30 days of first admission.

### Data analysis

Continuous data are summarized using median and interquartile range (IQR), and categorical data are summarized as number and percentage. Continuous data were compared between the patient cohort and the whole of England using Mann–Whitney *U* tests. Geospatial heat mapping was undertaken using Maptive online software (Maptive, San Francisco, California, USA; https://www.maptive.com).

## Results

### Patient characteristics

**Fig. 1:**
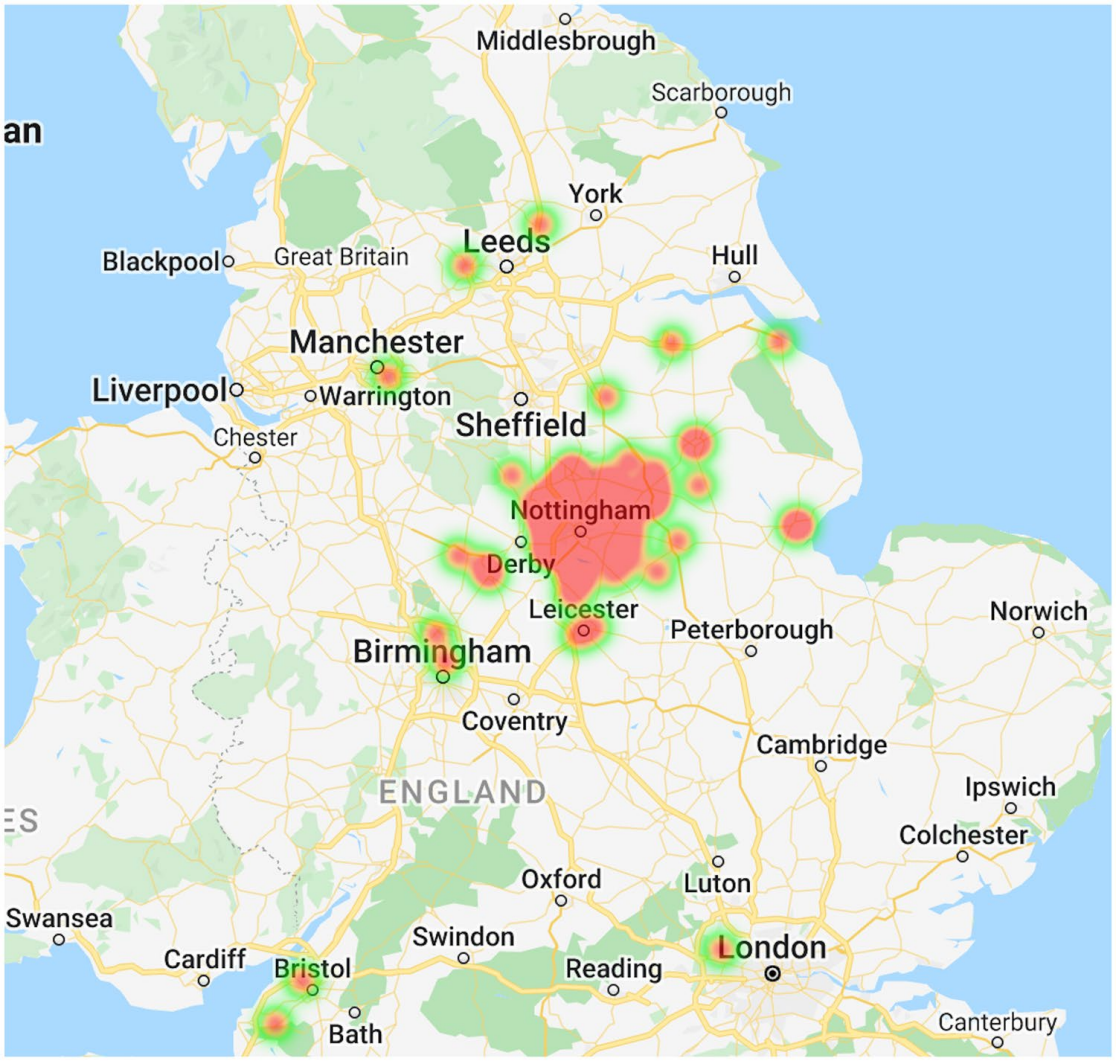
Geospatial heat mapping of home addresses of study patients. The colours red, yellow and green indicate density of patients as dense, medium and light respectively

There were 143 patient episodes identified and 2 were excluded due to incorrect coding. Two patients had two presentations during the study period. This left 139 patients in the study cohort, with a total of 141 presentations. Most patients were male [133/139 (96%)]. Patient characteristics are summarized in Table 1. Most patients were from Nottinghamshire, with some patients from surrounding cities within England. Geospatial distribution of patients is illustrated in Fig. 1. The distribution of timings of injury showed a peak during the evening period that coincides with the end of school and early evening (15:00 to 19:00; Fig. 2). 56/141 (40%) injuries occurred in the night (from 20:00 to 08:00). Patients had a median of 1 (IQR 1–2; range 1–12) stab wounds.

**Table 1:** Study patient characteristics

Patient characteristic	Summary data (*N* = 139)
Age, median (IQR)	16 (15–17)
Male gender, *n* (%)	133 (96)
Ethnic minority, *n* (%)	41 (29)
Stab wounds, median (IQR	1 (1–2)
Regions of injury*, *n* (%)	
Limbs	75 (53)
Head and neck	22 (16)
Chest	40 (28)
Abdomen	33 (23)
Buttocks	14 (10)
Treatments, *n* (%)	
TXA	35 (25)
Blood transfusion	21 (15)
Tourniquet	3 (2)
Hemostatic agent	4 (3)
Surgery	45 (32)
Outcomes, *n* (%)	
Mortality	2 (1)
Re-admitted within 30 days	12 (9)

*IQR* inter-quartile range, *TXA* tranexamic acid

*Denominator for regions of injury is all presentations (*N* = 141)

**Fig. 2:**
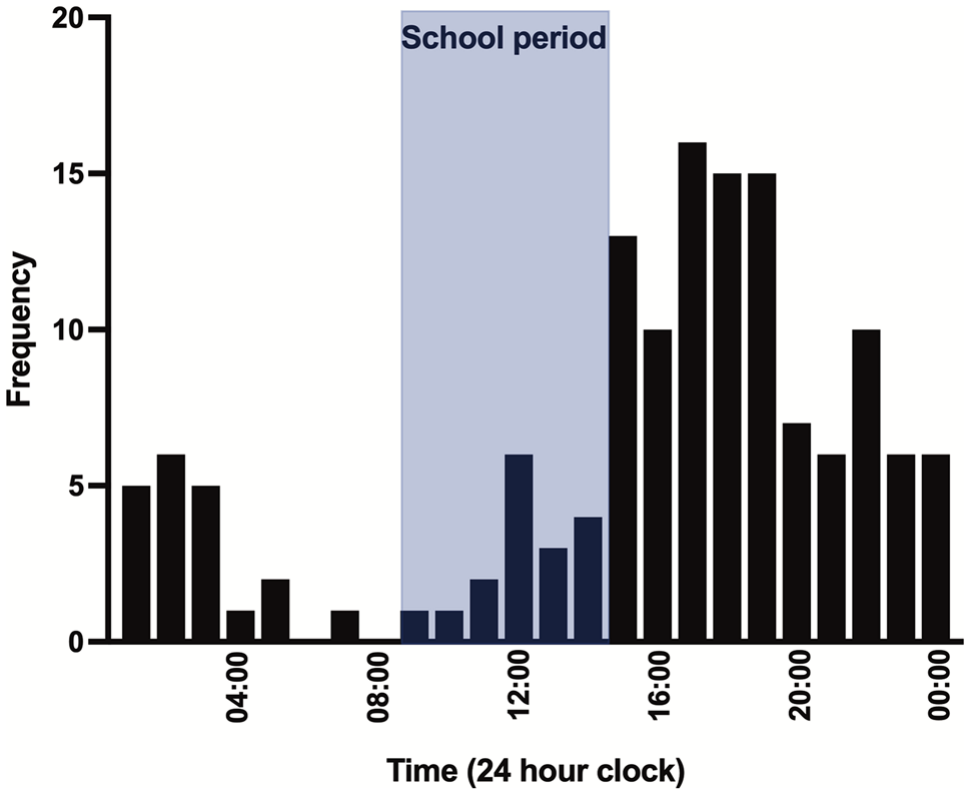
Timings of stabbing injuries for the study cohort, with indication of the approximate school period of 09:00 to 15:00

### Management of injuries

Fifty-eight (41%) of the presentations triggered a pre-hospital trauma alert for the attention of the Major Trauma team according to their physiology or mechanism of injury. There were 4/141 (3%) knife injuries managed with the haemostatic agent Celox (Medtrade Products Ltd, UK), 3/141 (2%) were managed with a tourniquet and 35/141 (25%) given tranexamic acid (Table 1). Blood product transfusion was required for 21/141 (15%) of patient episodes [median 3 (IQR 3–8) units of red blood cells and 1 (IQR 0–4) units of fresh frozen plasma]. For 74/141 (52%) patient episodes, the patient was discharged home directly from ED after their wounds were treated, with a median time of 227 (IQR 180–313) minutes in ED. The remainder [67/141 (48%)] resulted in admission to hospital. For 49/141 (35%) of the patient presentations, the patients had computed tomography (CT) imaging whilst in the ED. There were 45/141 (32%) patient episodes where surgery was required, with a median number of operations of 1 (IQR 1–1; range 1–3).

### Socioeconomic deprivation

**Fig. 3:**
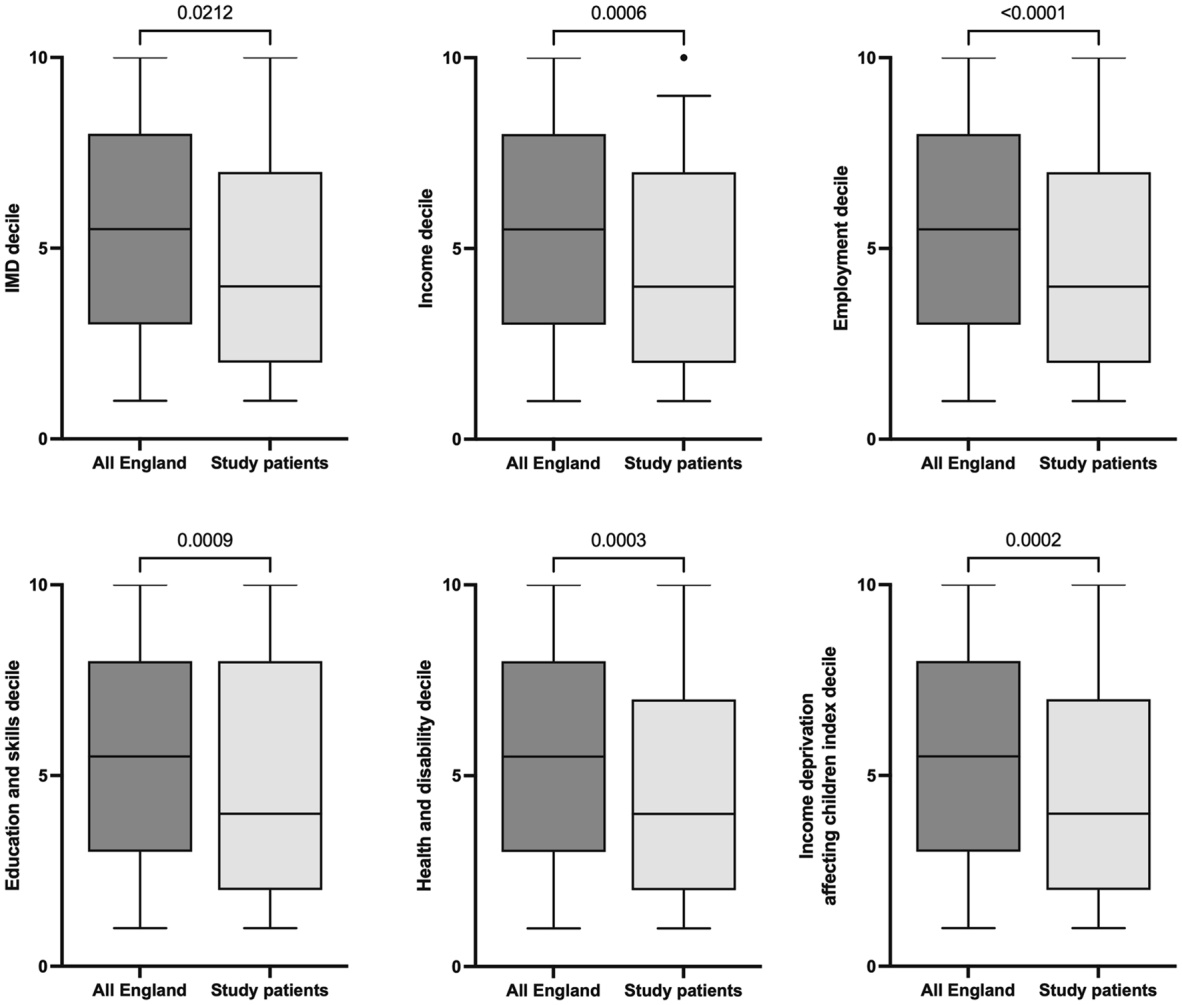
Indices of socioeconomic deprivation compared between patients and the rest of England

Figure 3 summarizes the indices of socioeconomic deprivation parameters for study patients compared to all of England. Study patients lived in areas with worse deciles of socioeconomic status, including IMD, income, employment, education and skills, health deprivation and disability and IDACI (Fig. 3). However, there were no significant differences between the study cohort and the rest of England for deciles in crime, barriers to housing and services or living environment.

### Outcomes

There were 2 patients who died during the study period, and therefore no risk factors could be identified for mortality due to low numbers. One patient died from further knife trauma in adulthood after the study period (aged 20) and 3 patients presented again with knife injuries in adulthood.

When those patients who were re-admitted within 30 days were compared to the remainder of the study cohort, there were no significant differences in socioeconomic parameters according to IMD [4 (IQR 1–7) vs 5 (IQR 2–7), respectively; *p* = 0.281], income [3 (IQR 1–5) vs 4 (IQR 2–7), respectively; *p* = 0.122], employment [3 (IQR 1–4) vs 4 (IQR 2–7), respectively; *p* = 0.111], education and skills [3 (IQR 1–7) vs 4 (IQR 2–8), respectively; *p* = 0.411], health deprivation and disability [4 (IQR 2–7) vs 4 (IQR 2–7), respectively; *p* = 0.698], crime [4 (IQR 3–8) vs 5 (IQR 3–9), respectively; *p* = 0.725], barriers to housing and services [7 (IQR 3–9) vs 6 (IQR 3–8) respectively; *p* = 0.925], living environment [5 (IQR 3–6) vs 6 (IQR 4–8), respectively; *p* = 0.169], or IDACI [3 (IQR 1–7) vs 4 (IQR 2–7), respectively; *p* = 0.191].

## Discussion

The main finding from the current study is that in a sample of 139 children presenting to hospital after being victims of knife crime over a 5-year period, patients were more likely to come from areas of socioeconomic deprivation when compared with the rest of England. Specific domains of deprivation included income (including income affecting children), employment, education and skills and health. Injury by knife violence did not appear to be a product of overall higher crime rates since there was no significant difference in the crime index between study patients and the rest of England. This finding suggests that the risk factors for vulnerability to violence are not simply higher crime rates but are more complex and multifactorial, relating to overall social and financial well-being.

Other public health discussions of knife crime have focussed on gang membership and how to reduce gang-related violence [13, 14]. It is likely that membership in gangs, violence and social deprivation all go hand in hand and therefore measures to address these must be multifactorial and community-focussed [15–17]. However, high quality studies that address youth gang prevention are sparse, and most evidence is based on the observational data only [18]. We were not able to analyse gang membership within the current study cohort, but it is likely that this may just be a consequence of the same complex socioeconomic factors that have been reported here.

Some solutions have been suggested in the efforts to reduce knife crime amongst younger people, including embedding youth workers within the ED of hospitals using the concept of the “teachable moment” [19, 20]. However, such interventions have had mixed results, with some showing limited success [21] and others showing some promise [22, 23]. Intervention after injury is likely to be insufficient as a public health measure if the socioeconomic risk factors identified in the current study are not also addressed. Such an approach would require funding and long-term strategy, but a recent UK report has shown that there has been a decrease in funding for youth services over the last decade [24]. The recent Home Office funding settlement for the Violence Reduction Units [25] may go a little way to redress this and provide longer term focus on the socioeconomic factors, which may facilitate a more effective longer term strategy. Data from the current study supports that hypothesis, but further investigations would be required to test it.

Knife violence within the current study cohort was less common during school hours and peaked in the early evening after the end of school. This is likely to be a consequence of the school age of the study cohort (with a median age of 16 years) and is in keeping with the findings of previous investigations of stabbings in young people in the UK [26]. There are some reports in the literature that propose structured after-school activities [27] including sports and leisure interests [28] to prevent violence in vulnerable groups. These kinds of activities may be community interventions that deserve further investigation. Strengthening engagement within school hours and before dismissal may also help prevent violence amongst younger people [29]. Within the catchment area of this study’s location there is an ongoing effort to reduce violence amongst young people by the National Youth Agency, supported by the Nottingham and Nottinghamshire Violence Reduction Unit and the Office of the Police and Crime Commissioner. Activities are designed to provide a place of safety, opportunities and connections to reduce the risk of violence [30].

In the UK, knife crime is far more prevalent than gun crime, and the latter is relatively rare compared with other similar nations such as the USA. This is most likely due to limited access to firearms secondary to relatively strict legislation, and an outright ban on all handguns. Injuries due to firearms were not investigated in the current study, but this and other modes of violence warrant further investigation in the context of socioeconomic factors. Knives are likely to be the weapon of choice for violence in the UK due to their easy accessibility and complex multifactorial influences on knife-carrying culture that include perceived benefits felt by carriers [31, 32].

### Limitations

The current study is observational and based on analysis of a database. It is therefore at risk of selection bias and missed patients. We are unable to prove causality between the socioeconomic deprivation and injury from knife crime, but instead can formulate hypotheses based on the compelling associations detected. The indices of deprivation were taken from an analysis conducted in 2019 but have been used as a marker for the study period of 2016–2022. However, this represents the most recent update to the governmental data, which was previously updated in 2015 prior to this iteration. The data are therefore considered to be a reasonable representation of the indices for the entire study period. None of the current study patients had self-inflicted injuries, but such a subgroup of patients may warrant further investigation to determine whether there are similar associations.

## Conclusion

Of 139 children who presented to a Major Trauma Centre in England in the last 5 years with injuries related to knife crime, patients were more likely to have a poor socioeconomic status compared to the rest of England in terms of income, education, health and wellbeing. These associations appear to be independent of overall levels of crime. Exposure to violence may be a consequence of these social and financial circumstances and focussed efforts to address socioeconomic disparities should be a priority as a public health measure for vulnerable children. Further investigations of the overall effects of such a strategy are warranted.

## Data Availability

Data are available from the authors upon reasonable request.
